# The Efficacy and Safety of Glibenclamide in Improving Cerebral Edema and Neurological Outcomes in Stroke: a GRADE-Evaluated Systematic Review and Meta-analysis with Subgroup Analysis

**DOI:** 10.1007/s12028-025-02311-3

**Published:** 2025-07-08

**Authors:** Hazem E. Mohammed, Mohamed E. Haseeb, Zeyad Bady, Mohamed Nasser, Mostafa Meshref

**Affiliations:** 1 Faculty of Medicine, Assiut University, Assiut, Egypt; 2 Faculty of Medicine, Minia University, Minia, Egypt; 3 Medical Research Group of Egypt, Cairo, Egypt; 4 Department of Neurology, Faculty of Medicine, Al-Azhar University, Cairo, Egypt

**Keywords:** Stroke, Glibenclamide, Glyburide, Ischemic stroke, Subarachnoid hemorrhage, Intracerebral hemorrhage, Systematic review, Meta-analysis

## Abstract

**Background:**

Stroke is a significant cause of morbidity and mortality worldwide, with cerebral edema being a major complication. Glibenclamide, a SUR1-TRPM4 channel inhibitor, has been proposed to reduce cerebral edema, but its clinical efficacy remains uncertain. This meta-analysis aimed to evaluate the efficacy and safety of glibenclamide in patients with stroke, including acute ischemic stroke, acute subarachnoid hemorrhage, and intracerebral hemorrhage.

**Methods:**

A comprehensive literature search was conducted in PubMed, Web of Science, and Scopus up to January 2025. The primary efficacy outcomes included excellent (modified Rankin Scale [mRS] score 0–1) and good (mRS score 0–2) functional outcomes at 90 days. Safety outcomes included the incidence of hypoglycemia and decompressive craniectomy. The quality of evidence was assessed using the Grading of Recommendations Assessment, Development, and Evaluation approach.

**Results:**

Ten and eight randomized controlled trials (RCTs) were included in our qualitative and quantitative analysis, respectively, encompassing 1,691 participants aged 18 to 85. No significant difference was observed between the glibenclamide and control groups regarding excellent functional outcome (risk ratio [RR] 1.10, 95% confidence interval [CI] 0.92–1.32, *P* = 0.29) and good functional outcome (RR 1.07, 95% CI 0.96–1.18, *P* = 0.22). Safety analysis revealed no significant increase in serious adverse events (RR 1.11, 95% CI 1.00–1.23, *P* = 0.06). Notably, hypoglycemia incidence after sensitivity analysis was higher in the glibenclamide group (RR 4.56, 95% CI 2.07–10.03, *P* = 0.0002).

**Conclusions:**

Glibenclamide did not significantly improve functional outcomes or reduce mortality in stroke patients but was associated with a higher incidence of hypoglycemia. Further well-designed RCTs are needed to clarify its therapeutic role and optimize safety protocols.

*Clinical trial registration*: PROSPERO registration number: CRD420251008350.

**Supplementary Information:**

The online version contains supplementary material available at 10.1007/s12028-025-02311-3.

## Introduction

Stroke is among the most significant problems facing the world today. Stroke can be diagnosed as ischemic stroke, intracranial hemorrhage, or subarachnoid hemorrhage [1]. Approximately 1.7 million patients die from stroke each year; 87% of these deaths are primary ischemic infarctions, 10% are hemorrhagic, and 3% are subarachnoid hemorrhages. Stroke is the most common type of cerebrovascular accident, the second major cause of mortality, and a major cause of disability worldwide [2, 3].

The incidence of stroke increases rapidly with age, doubling every decade after the age of 55. The annual incidence of stroke is 30 to 120 per 100,000 in adults aged 35 to 44 and 670 to 970 per 100,000 in those aged 65 to 74 [3]. Although stroke in children is much less common than in adults, sickle cell anemia is the most common cause of stroke in children [3]. Besides, subarachnoid hemorrhage is a serious form of stroke that affects about 600,000 individuals a year and may be caused by a ruptured aneurysm. Thirty percent of these individuals may experience function impairment, 20% may experience adverse outcomes, and 50% of these patients pass away from subarachnoid hemorrhage [4].

Cerebral edema is one of the most serious stroke complications that can result in cerebral herniation, intracranial pressure, or diminished neurological function [5–8]. In addition to being the primary risk factor for a poor prognosis in stroke, it can also result in death rates of more than 80% if not properly and rapidly treated [6, 9–11]. The goal of treatment is to reduce intracranial pressure and relieve any neurological symptoms or serious consequences that arise from this edema [7].

Because osmotic diuretics such as mannitol or hypertonic saline reduce the amount of water in brain tissue, they are currently the most widely used treatment for cerebral edema. However, their effectiveness in preventing these serious events is limited, and if misused or in excess, they can also result in kidney damage and electrolyte imbalance [6, 7].

Decompressive hemicraniectomy is a surgical intervention commonly used to reduce intracranial pressure due to cerebral edema and has shown a better result than osmotic diuresis in terms of intracranial pressure reduction if it is performed within the first 48 h and on patients younger than 60. However, if it is performed after 72 h or on patients older than 60, there is a significant risk of long-term comorbidity. Other limitations of the intervention, despite its invasive nature, include its dependence on the Glasgow Coma Scale (GCS)  score [8, 10, 12, 13].

As a result, there is a knowledge gap in the medical treatment of cerebral edema. Although some studies have attempted to prevent and treat cerebral edema using various mechanisms, such as AQP4 blockers, MMP9 inhibitors, and ion channel blockers such as NKCC1 and NHE, no human trials have been conducted for these mechanisms [6]. Glibenclamide, or glyburide, a SUR1 inhibitor commonly used by patients with type 2 diabetes, has been repurposed as an inhibitor of SUR1-TRPM4. Following  central nervous system (CNS)  injury, the SUR1 regulatory subunit is increased and combines with the TRPM4 channel to create a nonselective cation channel, which increases sodium influx. As a result, osmotic water moves into the cells, causing cytotoxic edema. By inhibiting SUR1, glibenclamide effectively blocks the SUR1-TRPM4 channel, thereby reducing sodium influx and subsequent water accumulation [8, 11, 14]. Glibenclamide was the only medication tested in human clinical trials, such as the GAMES-RP trial, that directly targets molecular pathways implicated in the development of cerebral edema [10]. CHARM, a recent phase III study of glibenclamide in large hemisphere stroke for cerebral edema, did not show statistically significant improvements in functional outcomes [11]. Because many trials have evaluated glibenclamide efficacy and safety, we planned to conduct this systemic review and meta-analysis. We aim to provide a comprehensive analysis, including all published randomized controlled trials (RCTs) up to this study date. Furthermore, we will provide an assessment of both certainty of evidence, using the Grading of Recommendations Assessment, Development, and Evaluation (GRADE) approach, and publication bias.

## Methods

### Protocol and Registration

This meta-analysis was conducted according to the Preferred Reporting Items for Systematic Reviews and Meta-Analyses (PRISMA) guidelines [15]. The study protocol was registered in PROSPERO (CRD420251008350).

### Eligibility Criteria

We included the studies that met the following criteria: (1) RCTs, (2) human studies, (3) studies written in English, (4) studies including patients aged 18 or older with any stroke type (e.g., acute ischemic stroke, subarachnoid hemorrhage, or intracerebral hemorrhage [ICH]), and (5) studies with glibenclamide as the intervention and placebo or standard care as the control. Meanwhile, studies written in languages other than English, single-arm trials, observational studies, animal studies, case reports, reviews, and conference abstracts were excluded.

### Information Sources and Search Strategy

A comprehensive search was conducted in the following electronic databases from inception to February 2025: PubMed, Web of Science, and Scopus. The search strategy can be found in Supplementary Table 1.

### Study Selection and Data Extraction

After conducting the search strategy, two authors blindly screened studies using Rayyan online software [16]. We began with title and abstract screening and then screened the full text. A third reviewer was consulted to resolve any conflict between the two authors regarding the inclusion decision. Two authors extracted data independently on an online Excel sheet for feasibility. The online sheet was divided into study characteristics, population baseline characteristics, and outcome measures data. Study characteristics included study name and year, sample size, design, location and duration of treatment, study population, and key findings. Population baseline characteristics included sample size, age, sex, race, ethnicity, baseline National Institute of Health Stroke Scale score, percentage of patients receiving recombinant tissue plasminogen activator, and relevant medical history. Outcome measures involved the following:

1. Efficacy outcomes were as follows:

- Functional outcomes were defined by the modified Rankin Scale (mRS): a 7-point scale ranging from 0 (no symptoms) to 6 (death), with higher scores indicating more significant disability. We reported the results as the percentage of patients achieving mRS scores of 0–1, 0–2, 3–5, and 6, along with the mean mRS score at day 90. If studies presented mRS data differently, we standardized the reporting format to ensure consistency across analyses. Functional outcomes were classified as excellent functional outcome (mRS score of 0–1), good functional outcome (mRS score of 0–2), and poor functional outcome (mRS score of 3–5) at 90 days.
- Mean midline shift at 72 h: the lateral displacement (mm) of midline structures on axial brain imaging, assessed at the level of the septum pellucidum or pineal gland.

2. Safety: Side effects included hypoglycemia, hydrocephalus, or decompressive craniotomy, in addition to cardiovascular adverse events.

### Risk of Bias Assessment

Two reviewers independently evaluated the risk of bias in the included RCTs using the Cochrane Risk of Bias tool 2.0 (RoB 2) [17]. The assessed domains included the randomization process bias, bias due to deviations from intended interventions, missing outcome data bias, bias in the measurement of the outcome, and bias in the selection of the reported result. Any disagreements were resolved through discussion or by consulting a third reviewer.

### Statistical Analysis

Review Manager 5.4 software (RevMan) was used for the statistical analysis [18]. We used RevMan to generate forest plots of both continuous and dichotomous outcomes. A random-effect model was considered in all outcomes due to the relatively small population. The analysis was based on a mean difference (MD) and standard deviation (SD) for continuous outcomes and on risk ratio (RR) with 95% confidence interval (CI) for dichotomous outcomes. A statistically significant *P* value was approved if it was < 0.05. Using the Higgins score (*I*^2^), the heterogeneity of the included studies was evaluated; *I*^2^ values ≥ 50% were indicative of high heterogeneity. Adverse events were reported as the number of events per study arm and pooled as an RR. A chi-square *P* value less than 0.1 was considered significant heterogeneity [19]. Furthermore, a sensitivity analysis was conducted to assess the heterogeneity and robustness of the results whenever possible.

### Sensitivity and Subgroup Analysis

Sensitivity analysis is a logical approach to test the robustness of evidence by excluding studies that are more likely to introduce heterogeneity to the pooled effect estimate [20]. We conducted a sensitivity analysis to resolve and explore heterogeneity in heterogeneous outcomes. A primary analysis of all the studies was performed, followed by a secondary one with a sensitivity analysis. Additionally, we conducted a subgroup analysis of our primary outcomes according to two criteria: the control group (placebo versus standard care) and type of stroke (ischemic, subarachnoid hemorrhage, and ICH).

### Certainty of Evidence

The certainty of evidence for each outcome was assessed using the GRADE approach, considering the risk of bias, inconsistency, indirectness, imprecision, and publication bias [21, 22].

### Publication Bias

The publication bias was assessed using the Doi plot and the Luis Furuya–Kanamori asymmetry index (LFK index). Asymmetry of the plots is a reflection of publication bias, whereas symmetry is a reflection of bias absence. No asymmetry, mild asymmetry, and severe asymmetry are indicated by LFK index values of ≤  ± 1, >  ± 1, but ≤  ± 2 and >  ± 2, respectively [23]. We created Doi plots for our main findings and performed publication bias analysis using the MetaXL Version 5.3 add-in for Microsoft Excel [24].

## Results

### Literature Search

**Fig. 1:**
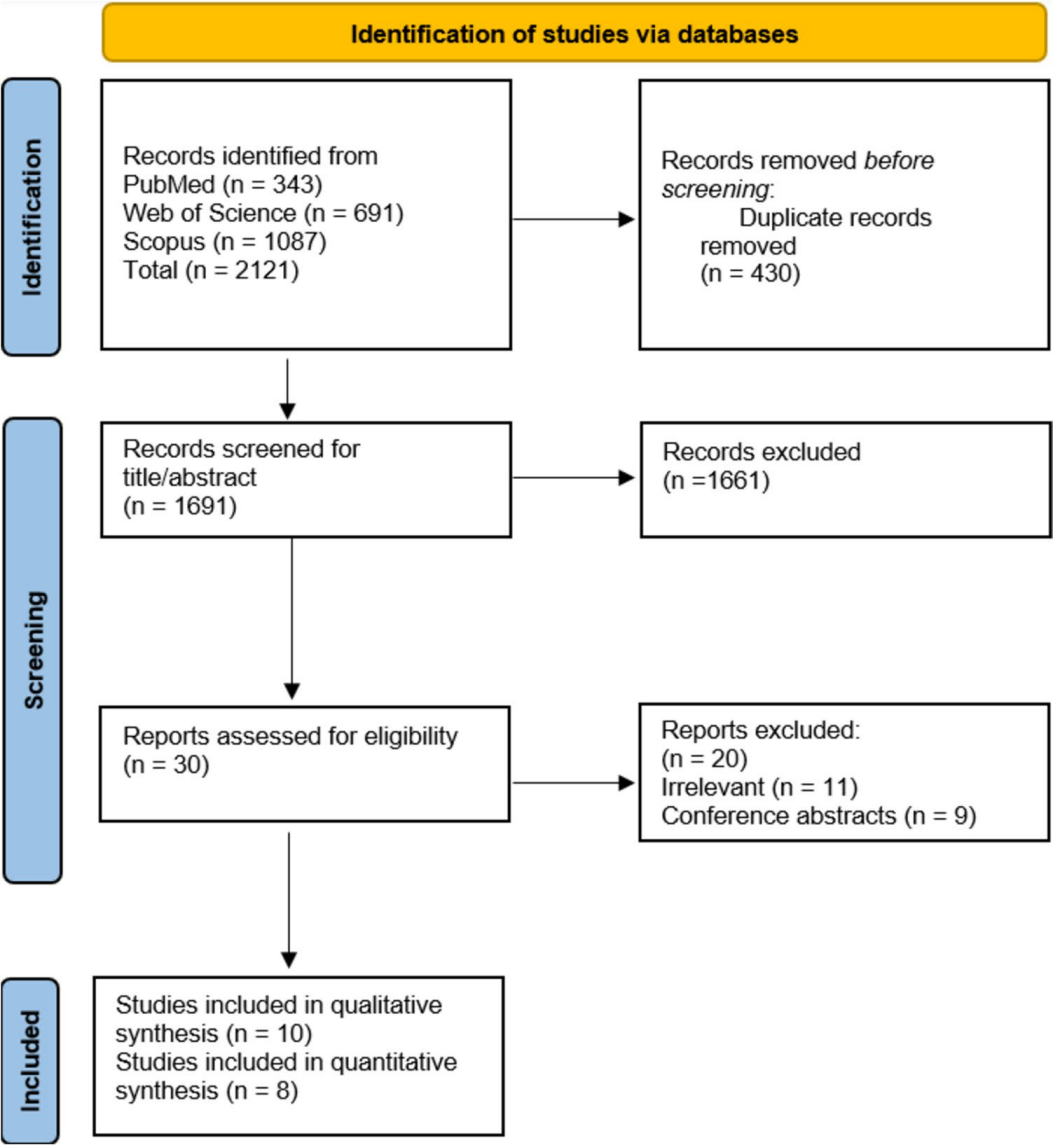
Preferred Reporting Items for Systematic Reviews and Meta-Analyses flow diagram

After conducting our search strategy, we identified 2,121 records. After removing 430 duplicate records, 1,691 unique records remained. These records underwent title and abstract screening, resulting in the exclusion of 1,661 records. Subsequently, 30 reports were assessed for full-text eligibility, and 20 were excluded for not meeting the inclusion criteria. Finally, ten studies were included in the qualitative synthesis [1, 2, 4, 8–11, 14, 25, 26], and eight were included in the quantitative synthesis [1, 2, 4, 8–11, 26]. The PRISMA flow diagram is presented in Fig. 1.

### Study and Population Characteristics

We examined ten studies conducted from 2016 to 2024. These studies focused on people aged 18 to 85 who had an acute ischemic stroke, acute subarachnoid hemorrhage, or ICH. The sample sizes ranged between 45 and 431 participants. The age of participants was between 50 and 63 years. Most participants were male, making up between 46 and 68% of the groups. Common health issues among participants included hypertension, diabetes, and coronary artery disease, with hypertension being the most common. All studies assessed the effectiveness of glibenclamide compared to a placebo or standard care. Studies characteristics are shown in Table 1, and baseline characteristics of the participants are represented in Table 2.

**Table 1:** Included studies characteristics

Study	Sample Size	Design	Location and Duration	Study Population	Control	Administration of Glibenclamide	Key Findings
Sheth et al. [11]	431 Glibenclamide:217 Placebo:214	A phase 3, double-blind, placebo-controlled randomized trial	The trial was conducted at 143 acute stroke centers in 21 countries and lasted for 3 months	Patients aged 18 to 85 years, with acute ischemic stroke in at least the middle cerebral artery territory within 10 h of stroke onset, NIHSS Score of 10 or higher, and no premorbid disabilities	Placebo	Intravenous route	There was no difference in disability between glibenclamide, and placebo groups as assessed by the mRS at 90 days (P-value = 0·42). Regarding safety profile, serious adverse events were reported in 77% of the glibenclamide group and 68% of the placebo group
Lin et al. [2]	111 Glibenclamide:57 Control:54	A multicenter, prospective, randomized, controlled, open-label, blinded end-point clinical trial	The trial was conducted in Beijing Tiantan Hospital and Beijing Tongren Hospital and lasted 3 months	Patients aged 18 to 74 years, with radiology-confirmed acute subarachnoid hemorrhage within 48 h	Standard care	Oral route	There was no significant difference in the mRS score at 90 days between the glibenclamide and control groups (P-value = 0.681). The most common adverse events were bleeding and hydrocephalus. However, no significant difference between both groups was found (P-value > 0.05)
Feng et al. [1]	56 Glibenclamide: 28 Placebo:28	A randomized, double-blind, placebo-controlled clinical trial	The trial was conducted at a single center in China and lasted 6 months	Patients aged 18 years or older, with radiology-confirmed acute subarachnoid hemorrhage, underwent surgery within 72 h of admission and had a Hunt-Hess grade of 2 or higher	Placebo	Oral route	The mRS score at 3 and 6 months showed no difference between the glibenclamide and placebo groups with P-values = 0.996 and 0.890, respectively. The most common adverse events were pulmonary infection and liver and kidney dysfunction. However, no significant difference between both groups was observed with P-values = 0.086 and 0.789, respectively
Windlin et al. [25]	45 Glibenclamide: 23 Placebo:22	A randomized controlled trial	The trial was conducted at a neurocritical care unit in Brazil and lasted 21 days	Patients aged 18 to 70 years, with radiology-confirmed acute subarachnoid hemorrhage and aneurysmal origin, underwent clipping or coiling within 96 h of ictus	Placebo	Oral route	There was no significant difference in the proportion of patients achieving favorable outcomes, defined as an mRS score of 0–2, between the glibenclamide and placebo groups (P-value = 0.454). Regarding emotional aspects, the HADS and SPTSS scales showed no significant difference between both groups (P-value > 0.05)
Huang et al. [9]	272 Glibenclamide:142 Plcebo:130	A randomized, double-blind, placebo-controlled, parallel-group trial	The trial was conducted at 8 academic hospitals in China and lasted 3 months	Patients aged 18 to 74 years, with acute ischemic stroke, received IV rtPA within 4.5 h of symptom onset, with a symptomatic anterior circulation occlusion, and an NIHSS score of 4–25	Placebo	Oral route	There was no significant difference in the proportion of patients achieving an mRS score of 0–2 at 90 days between the glibenclamide and placebo groups (P-value = 0.96). The most common adverse outcomes were pulmonary infection and cardiac events. However, no significant difference between both groups was observed with P-values = 0.77 and 0.72, respectively
Zhao et al. 2022^26^	200 Glibenclamide: 99 placebo: 101	A multicentre, prospective, randomized, controlled open-label trial	The trial was conducted at 26 hospitals in China and lasted for 2 years with follow-up	Patients aged 18 years or older with primary hemorrhage in basal ganglia from 5 to 30 ml, with initial GCS 6 or more and with symptoms onset less than 72 h before admission	Standard care	Oral route	In the glibenclamide group, the incidence of a 90-day poor outcome (mRS > 3) was slightly lower than in the control group (20/99 [20.2%] vs. 30/101 [29.7%], absolute difference: 9.5%, 95% CI: -3.2% ~ 21.8%; P = 0.121). Glibenclamide administration did not significantly lower the probability of a bad outcome within 90 days (adjusted OR: 0.54, 95% CI: 0.24 ~ 1.20; P = 0.129)
Costa et al. [4]	78 glibenclamide: 38 placebo: 40	A double-blinded randomized control trial	The trial was conducted at Hospital das Clínicas, University of São Paulo, Brazil. and lasted for 21 days + 6 months follow-up period	Patients between 18 and 70 yearsthat diagnosed with SAH by CT or MRI and received definitive treatment within 96 h	Placebo	Oral route	There was no significant difference in functional outcome mRS after 6 months period of receiving glibenclamide between the glibenclamide and placebo groups. (ordinal analysis, unadjusted common OR 0.66 [95% CI 0.29–1.48], adjusted common OR 1.25 [95% CI 0.46–3.37]). Similar results were found for analyses considering the dichotomized 6-month mRS score (favorable score 0–2),
Eisenberg et al. [19]	29 glibenclamide:15placebo: 14	A double-blinded randomized control trial	The trial was conducted at (University of California, San Diego) and lasted for 30 months	Patients aged 18–75 years also, with GCS 4–8 or 9–14 and free of sedative or paralytic drugs and with documented closed traumatic brain injury	Placebo	Oral route	According to GOS-E at 90 days, three patients in the glibenclamide group were classified as vegetative or seriously impaired, although half of the patients recovered well. At 90 days, there was no significant difference between the groups when dichotomising for GOS-E ≤ 4 versus > 4 at (p = 0.32)
Kimberly [8]	77 Glibenclamide 41 placebo: 36	A double-blinded randomized control trial	The trial was conducted at 18 hospitals in the USA and lasted for 90 days	Patients aged 18–80 with large anterior hemispheric infarction for less than 10 h confirmed by diffusion-weighted image lesion volume of 82–300 centi-meter^3^	Placebo	Oral route	There is no significant difference between the glibenclamide and placebo groups in the frequency of hemorrhagic transformation (P-value = 0.91) or the incidence of malignant edema (P-value = 0.94). however, there is a reduced proportion of deaths attributed to cerebral edema in the glibenclamide group (P-value = 0.01) and also a lower rate in the NIHSS scale P-value = 0.043
Sheth et al. [10]	77 Glibenclamide 41 placebo: 36	A double-blinded randomized control trial	This trial was conducted at 18 hospitals in the USA and lasted for 90 days	Patients aged 18–80 with large anterior hemispheric infarction for less than 10 hoursconfirmed by diffusion-weighted image lesion volume of 82–300 cm^3^	placebo	Intravenous route	The primary clinical outcome mRS 0–4 without decompressive craniectomy did not differ between the glibenclamide and placebo groups (41% vs. 39%; adjusted P-value = 0·77). there was no significant difference in decompressive craniectomy or death by day 14, nor was there a the difference in lesional swelling or ipsilateral hemisphere swelling between the two groups between 72 and 96 h (centi-meter^3^)

NIHSS: National Institutes of Health Stroke Scale; mRS: Modified Rankin Scale; HADS: Hospital Anxiety and Depression Scale; SPTSS: Screen for Posttraumatic Stress Symptoms; OR: Odds Ratio; CI: Confidence Interval; GCS: Glasgow Coma Scale; GOS-E: Glasgow Outcome Scale – Extended

**Table 2:** Baseline characteristics of participants

Study	Groups (Number)	Age (years), mean (SD)	Sex, Male, N (%)	Race, N (%)				Ethnicity, N (%)		NIHSS score, mean (SD)	IV rtPA, N (%)	Medical history, N (%)				
				White	Black	Asian	Other	Hispanic	Non-Hispanic			Diabetes	HTN	Coronary artery disease	Ischaemic stroke or TIA	Atrial fibrillation
Sheth et al. 2024^11^	Glibenclamide (N = 217)	58 (9.5)	147 (68)	141 (65)	17 (8)	44 (20)	14 (6)	34 (16)	183 (84)	20 (2.24)	82 (38)	NA	NA	NA	NA	NA
	Control (N = 214)	58·7 (9)	141 (66)	131 (61)	19 (9)	44 (21)	12 (6)	29 (14)	185 (86)	20.3 (2.99)	84 (39)	NA	NA	NA	NA	NA
Lin et al. 2024^2^	Glibenclamide (N = 57)	57 (11.41)	28 (49)	NA	NA	NA	NA	NA	NA	NA	NA	6 (11)	35 (61)	NA	NA	NA
	Control (N = 54)	55.3 (11.04)	25 (46)	NA	NA	NA	NA	NA	NA	NA	NA	4 (7)	27 (50)	NA	NA	NA
Feng et al. 2024^1^	Glibenclamide (N = 28)	61.8 (11.6)	12 (42.9)	NA	NA	NA	NA	NA	NA	NA	NA	6 (21.4)	23 (82.1)	4 (14.3)	NA	NA
	Control (N = 28)	59.1 (12.6)	17 (60.7)	NA	NA	NA	NA	NA	NA	NA	NA	2 (7.1)	18 (64.3)	7 (25)	NA	NA
Windlin et al. 2024^25^	Glibenclamide (N = 23)	49.9 (11.9)	2 (8.7)	NA	NA	NA	NA	NA	NA	NA	NA	NA	NA	NA	NA	NA
	Control (N = 22)	50.0 (12.1)	10 (45.5)	NA	NA	NA	NA	NA	NA	NA	NA	NA	NA	NA	NA	NA
Huang et al. 2023^9^	Glibenclamide (N = 142)	61 (11)	103 (73)	NA	NA	NA	NA	NA	NA	8.67 (5.24)	NA	20 (14)	85 (60)	NA	19 (13)	13 (9)
	Control (N = 130)	61 (12)	91 (70)	NA	NA	NA	NA	NA	NA	8 (4.5)	NA	19 (15)	66 (51)	NA	22 (17)	11 (8)
Zhao et al. 2022^26^	Glibenclamide (N = 99)	56 (11)	67 (67.7)	NA	NA	NA	NA	NA	NA	7.33 (3.76)	NA	4 (4)	81 (81.8)	6 (6.1)	10 (10.1)	1 (1)
	Control (N = 101)	56 (10)	61 (60.4)	NA	NA	NA	NA	NA	NA	8 (6.1)	NA	9 (8.9)	87 (86.1)	11 (10.9)	12 (11.9)	2(2)
Costa et al. 2022^4^	Glibenclamide N (38)	53.6 (11.6)	6 (15.8)	21 (55.3)	8 (21.1)	NA	NA	NA	NA	NA	NA	NA	NA	NA	NA	NA
	Control N (40)	52.7 (11.3)	13 (32.6)	22 (55.0)	6 (15)	NA	NA	NA	NA	NA	NA	NA	NA	NA	NA	NA
Eisenberg et al. 2019^14^	Glibenclamide (N = 15)	NA	NA	NA	NA	NA	NA	NA	NA	NA	NA	NA	NA	NA	NA	NA
	Control (N = 14)	NA	NA	NA	NA	NA	NA	NA	NA	NA	NA	NA	NA	NA	NA	NA
Kimberly 2018^8^	Glibenclamide N (41)	58 (11)	25 (61)	35 (85)	4 (10)	2 (5)	NA	NA	NA	21 (8.45)	25 (61)	8 (20)	31 (76)	8 (20)	6 (15)	13 (32)
	Control N (36)	63 (9)	26 (72)	30 (83)	4 (11)	2 (6)	NA	NA	NA	20.3 (4.6)	22 (61)	7 (19)	24 (67)	4 (11)	4 (11)	14 (39)
Sheth et al. 2016^10^	Glibenclamide N (41)	58 (11)	25 (61)	35 (85)	4 (10)	2 (5)	NA	NA	NA	21 (8.45)	25 (61)	8 (20)	31 (76)	8 (20)	6 (15)	13 (32)
	Control n (36)	63 (9)	26 (72)	30 (83)	4 (11)	2 (6)	NA	NA	NA	20.3 (4.6)	22 (61)	7 (19)	24 (67)	4 (11)	4 (11)	14 (39)

NIHSS: National Institutes of Health Stroke Scale; IV rtPA: Intravenous Recombinant Tissue Plasminogen Activator; HTN: Hypertension; TIA: Transient Ischemic Attack; NA: Not available

### Quality Assessment

**Fig. 2:**
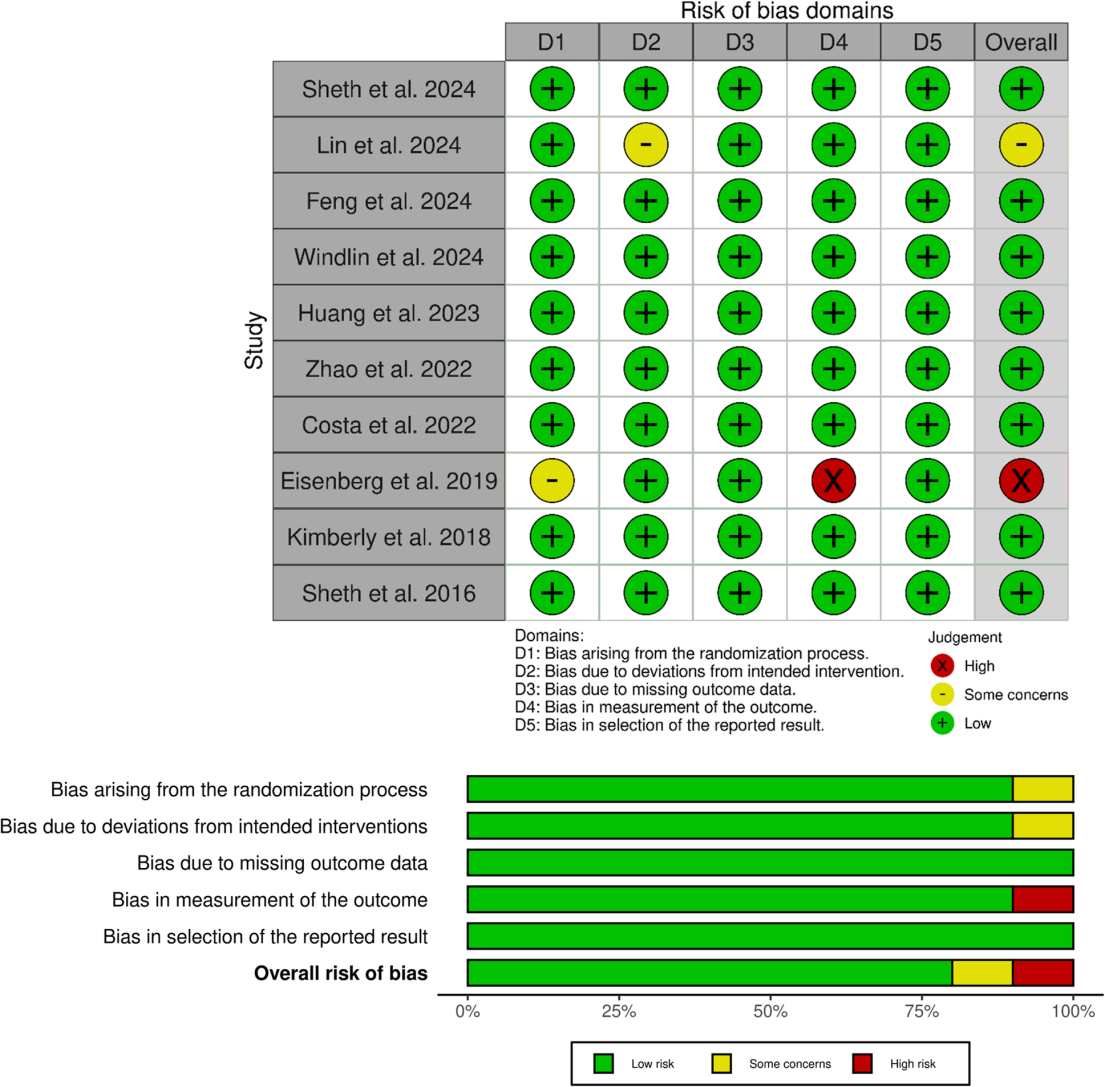
Risk of bias summary and graph

The risk of bias was evaluated using RoB 2. Of the ten included studies, eight were determined to have a low risk of bias across all assessed domains, and two studies showed some concerns or high risk in only one domain. The detailed risk of bias assessment is presented in Fig. 2.

### Efficacy of Glibenclamide

**Fig. 3:**
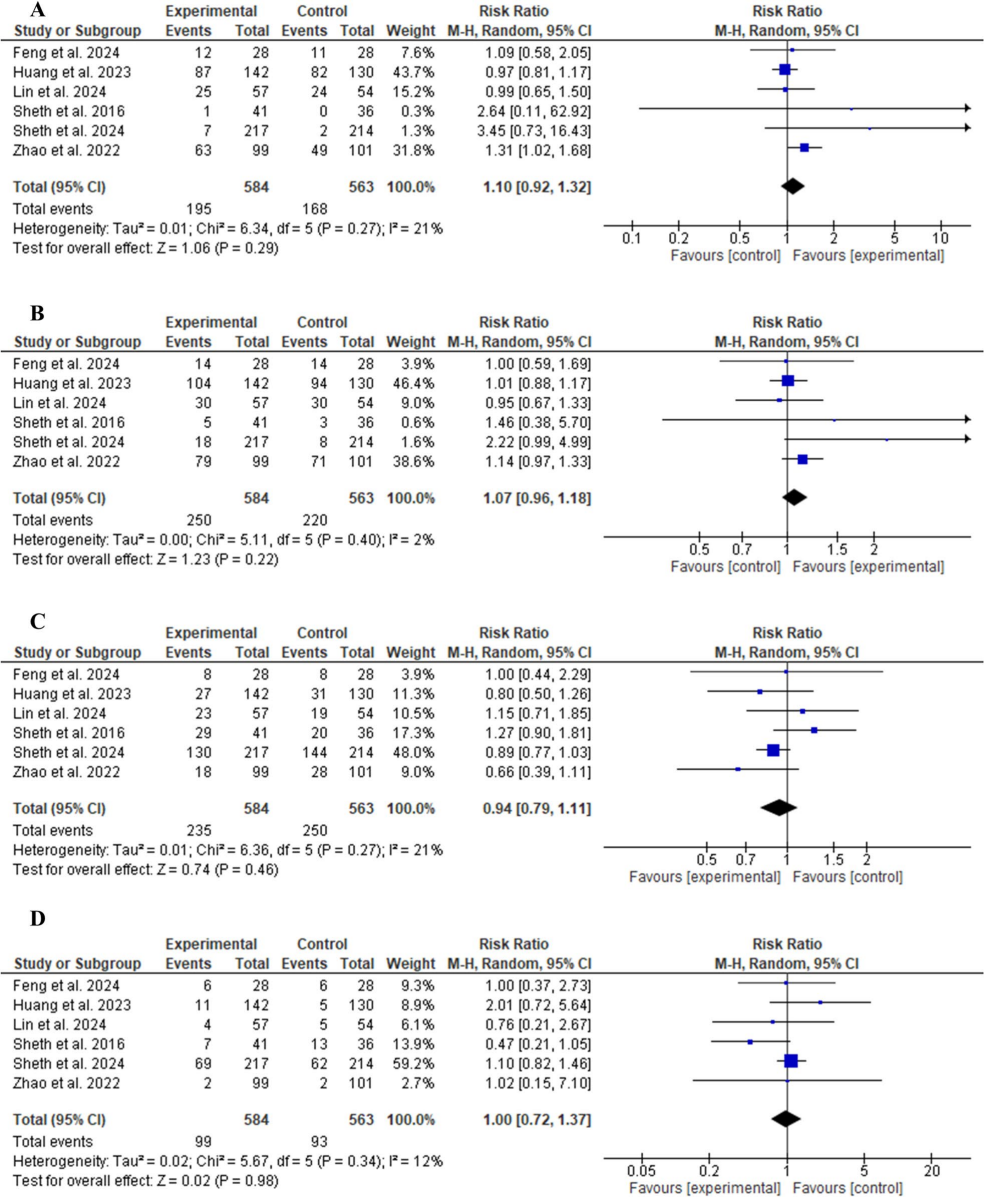
Comparison between glibenclamide and control groups in terms of excellent functional outcome (90-day mRS score of 0–1) (**a**), good functional outcome (90-day mRS score of 0–2) (**b**), poor functional outcome (90-day mRS score of 3–5) (**c**), and death (90-day mRS score of 6) (**d**). CI confidence interval, mRS modified Rankin Scale

**Fig. 4:**
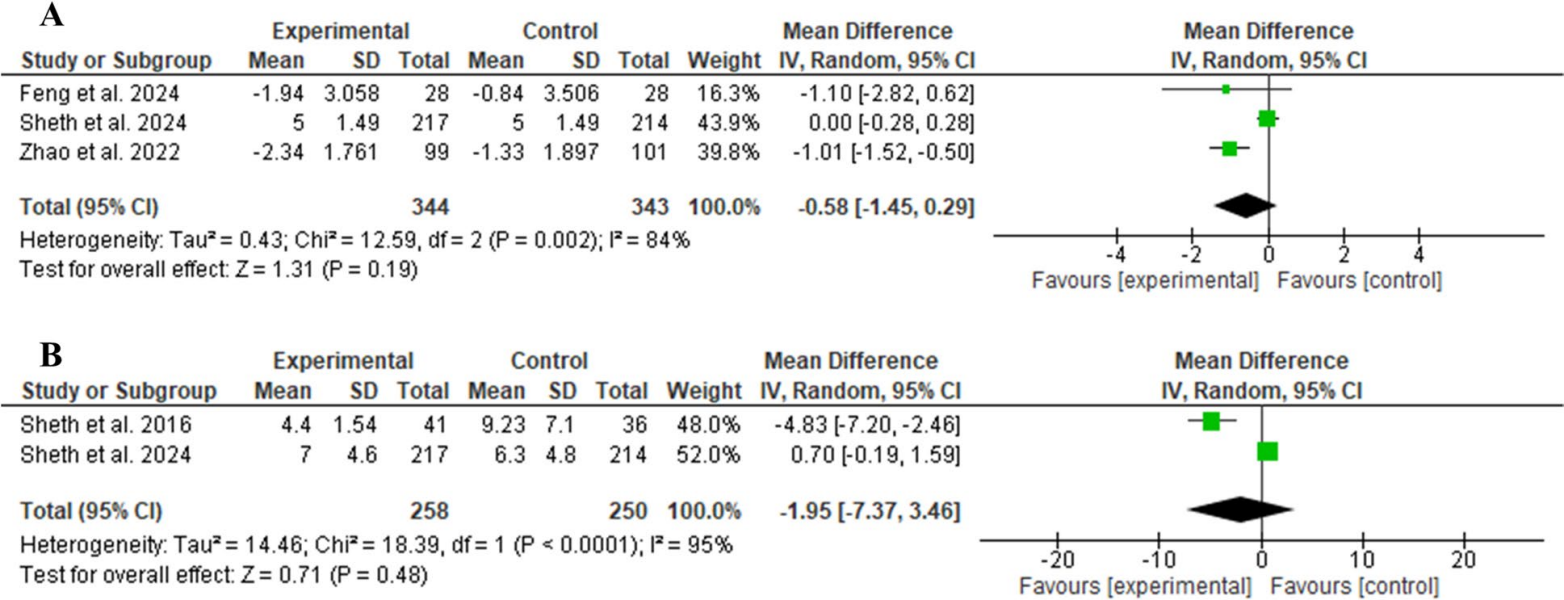
Comparison between glibenclamide and control groups in terms of mRS score at 90 days (**a**) and mean midline shift at 72 h (**b**). CI confidence interval, mRS modified Rankin Scale

The glibenclamide group did not show a statistically significant difference regarding the number of patients achieving excellent functional outcome (mRS score 0–1) compared to the control group (RR 1.10, 95% CI 0.92–1.32, *P* = 0.29, as shown in Fig. 3A). Similarly, glibenclamide did not exhibit a significant difference in terms of good functional outcome (mRS score 0–2) (RR 1.07, 95% CI 0.96–1.18, *P* = 0.22, as shown in Fig. 3B). For poor functional outcome (mRS score 3–5; RR 0.94, 95% CI 0.79–1.11, *P* = 0.46, as shown in Fig. 3C) and an mRS score of 6 (RR 1.00, 95% CI 0.72–1.37, *P* = 0.81, as shown in Fig. 3D), there were no significant differences in their incidence. Regarding the 90-day mRS score, there was no statistically significant difference between the two groups (MD − 0.58, 95% CI − 1.45 to 0.29, *P* = 0.19, as shown in Fig. 4A). The change in mean midline shift at 72 h was not statistically different between the two groups (MD − 1.95, 95% CI − 7.37 to 3.46, *P* = 0.48, as shown in Fig. 4B).

### Safety of Glibenclamide

**Fig. 5:**
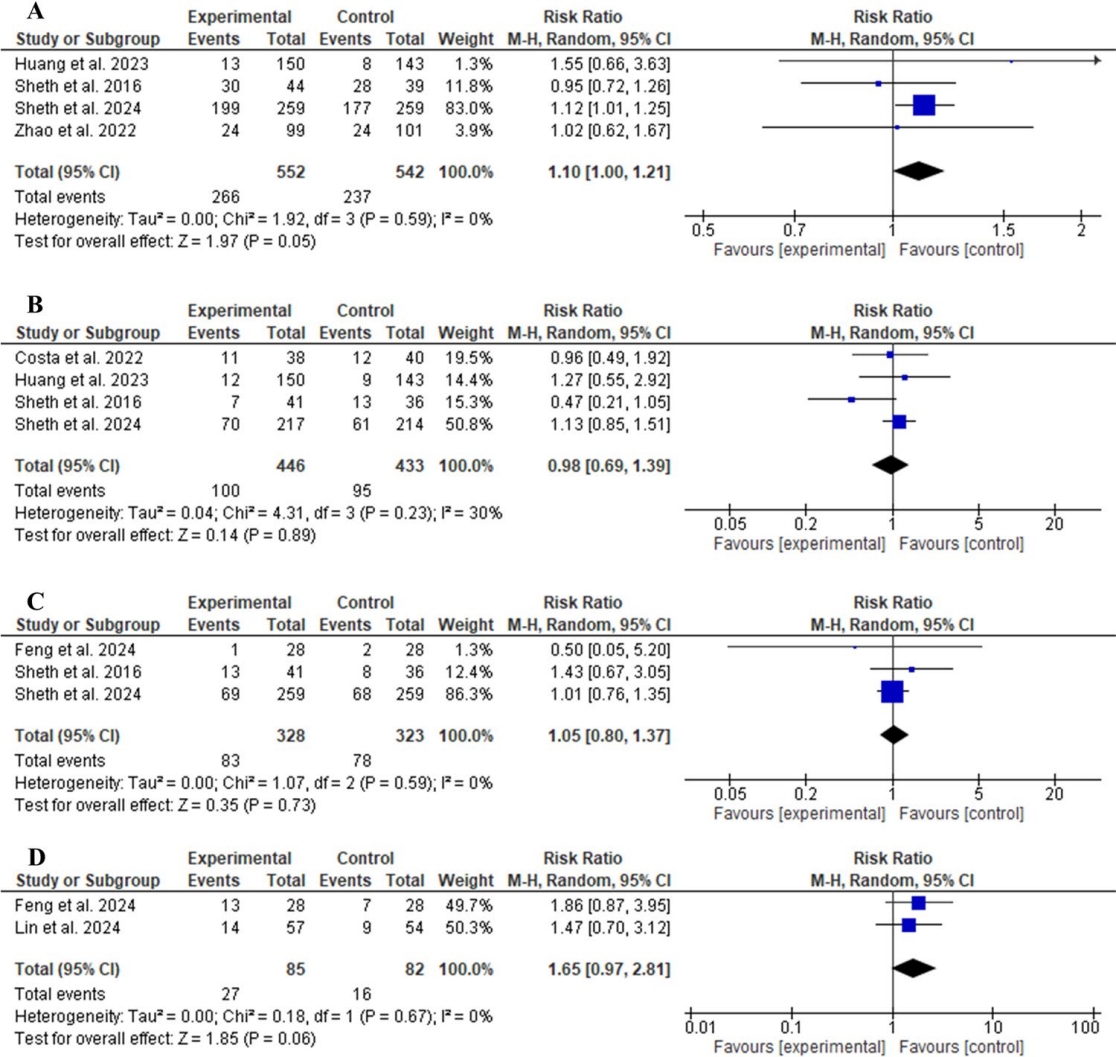
Comparison between glibenclamide and control groups in terms of any serious adverse events (**a**), 90-day mortality (**b**), decompressive craniotomy (**c**), and hydrocephalus (**d**). CI confidence interval

**Fig. 6:**
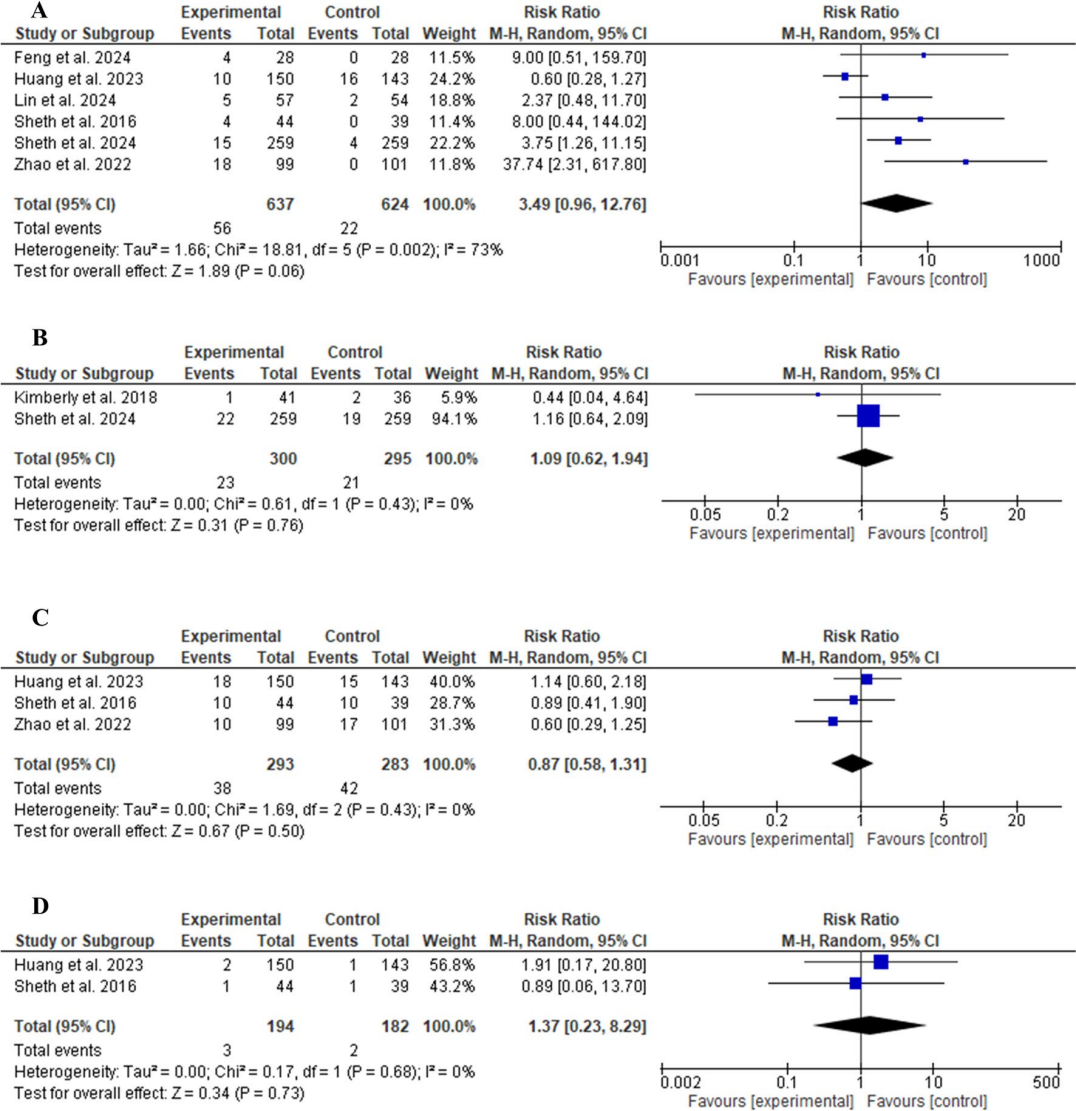
Comparison between glibenclamide and control groups in terms of hypoglycemia (**a**), parenchymal hematomas (**b**), cardiac events (**c**), and cardiac deaths (**d**). CI confidence interval

The safety profile of the glibenclamide group was comparable to that of the control group. No significant difference was detected in the risk of any serious adverse events (RR 1.10, 95% CI 1.00–1.21, *P* = 0.05, as shown in Fig. 5A). Regarding 90-day morality (RR 0.98, 95% CI 0.69–1.39, *P* = 0.89, as shown in Fig. 5B), decompressive craniotomy (RR 1.05, 95% CI 0.80–1.37, *P* = 0.73, as shown in Fig. 5C), and hydrocephalus (RR 1.65, 95% CI 0.97–2.81, *P* = 0.06, as shown in Fig. 5D), there was no significant difference in their risks. Similarly, no statistically significant differences were observed in the risk of hypoglycemia (RR 3.49, 95% CI 0.96–12.76, *P* = 0.06, as shown in Fig. 6A), parenchymal hematomas (RR 1.09, 95% CI 0.62–1.94, *P* = 0.76, as shown in Fig. 6B), cardiac events (RR 0.87, 95% CI 0.58–1.31, *P* = 0.50, as shown in Fig. 6C), and cardiac deaths (RR 1.37, 95% CI 0.23–8.29, *P* = 0.73, as shown in Fig. 6D).

### Sensitivity and Subgroup Analysis

Most of our outcomes were homogenous except for the mRS score at 90 days for efficacy outcomes (*I*^2^ = 84%) and hypoglycemia for safety outcomes (*I*^2^ = 73%). The mRS score at 90 days heterogeneity was best resolved by excluding the study by Sheth et al. [11] (*I*^2^ = 0%), interestingly yielding a statistically significant pooled estimate unlike what was before sensitivity analysis (MD − 1.02, 95% CI − 1.50 to − 0.53, *P* < 0.0001). The heterogeneity of hypoglycemia was resolved by excluding the study by Huang et al. [9], resulting in significant risk in the glibenclamide group (RR 4.56, 95% CI 2.07–10.03, *P* = 0.0002). The forest plots of the two aforementioned outcomes are demonstrated in Supplementary Fig. 1. We conducted a subgroup analysis for our primary functional outcomes according to the control group assigned in the included RCTs, whether it was placebo or standard treatment. No statistically significant subgroup difference has been detected at any of the functional outcomes, as show in in Supplementary Fig. 2. Regarding subgrouping according to type of stroke, no statistically significant subgroup difference existed at any of our primary functional outcomes, as show in in Supplementary Fig. 3.

### Publication Bias

**Fig. 7:**
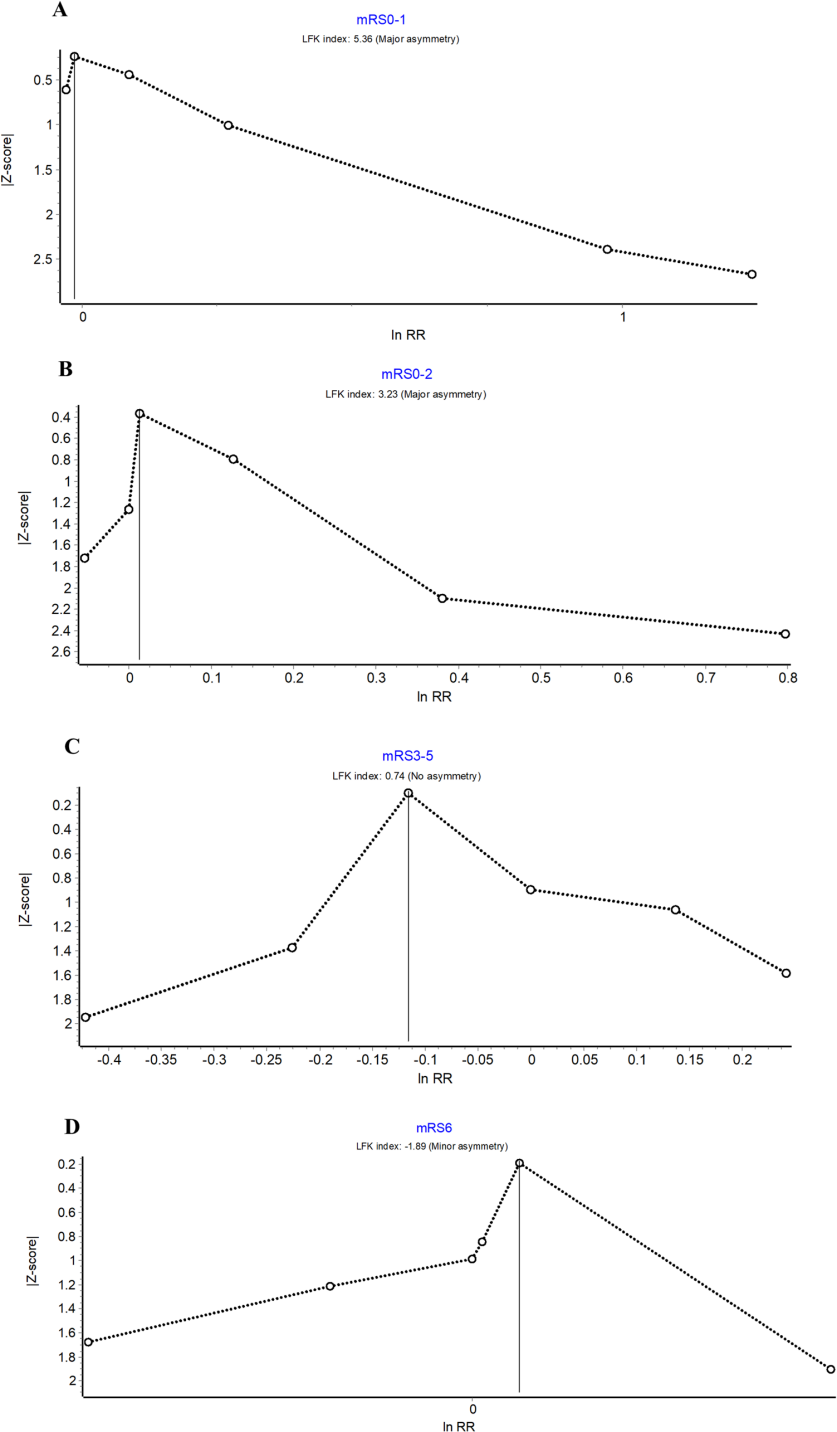
Publication bias with Doi plots and LFK index for excellent functional outcome (90-day mRS score of 0–1) (**a**), good functional outcome (90-day mRS score of 0–2) (**b**), poor functional outcome (90-day mRS score of 3–5) (**c**), and death (90-day mRS score of 6) (**d**). CI confidence interval, LFK Luis Furuya–Kanamori asymmetry, mRS modified Rankin Scale, RR risk ratio

Regarding publication bias, the Doi plot of excellent functional and good functional outcomes showed major asymmetry, with an LFK index of 5.36 and 3.23, respectively, as shown in Fig. 7, suggesting potential publication bias. However, poor functional outcomes demonstrated no asymmetry, as shown in Fig. 7.

### Quality of the Evidence

The quality of evidence regarding glibenclamide efficacy and safety in the most important and relevant outcomes versus control was assessed using GRADE. Regarding efficacy, the certainty about the results of patients attaining an mRS score of 0–1 and 0–2 was of low certainty. The certainty about the results of patients attaining an mRS score of 3–5 and 6 was of moderate certainty. Besides, the evidence regarding the mRS score assigned to patients 3 months after using glibenclamide was of low certainty due to imprecision and heterogeneity of clinical trials’ outcomes. The quality of evidence about safety was of moderate certainty due to an inconclusive pooled effect compared to control. Interestingly, the evidence regarding glibenclamide causing hypoglycemia was of low certainty because of the combined imprecision of the pooled effect estimate and the heterogeneity of clinical trials’ outcomes. The GRADE evaluation can be found in Table 3.

**Table 3:** Summary of findings and quality of evidence

Certainty assessment							Summary of findings				
Outcomes/ Number of patients (number of studies)	Risk of bias	Inconsistency	Indirectness	Imprecision	Publication bias	Overall certainty of evidence	Study event rates (%)		Relative effect (95% CI)	Anticipated absolute effects	
							With control	With intervention		Risk with control	Risk difference with intervention
90d mRS score of 0–1											
1147 (6 RCTs)	Not serious	Not serious	Not serious	Serious^b^	Publication bias strongly suspected by LFK index	⨁⨁◯◯ Low	168/563 (29.8%)	195/584 (33.4%)	**RR 1.10** ( 0.92to 1.32)	298 per 1000	**30 more per 1,000** (from 24 fewer to 95 more)
**90d mRS score of 0–2**											
1147 (6 RCTs)	Not serious	Not serious	Not serious	Serious^b^	Publication bias strongly suspected by LFK index	⨁⨁◯◯ Low	220/563 (39.1%)	250/584 (42.8%)	**RR 1.07** ( 0.96to 1.18)	391 per 1000	**27 more per 1,000** (from 16 fewer to 70 more)
**90d mRS score of 3–5**											
1147 (6 RCTs)	Not serious	Not serious	Not serious	Serious^b^	None	⨁⨁⨁◯ Moderate	250/563 (44.4%)	235/584 (40.2%)	**RR 0.94** ( 0.79to 1.11)	444 per 1000	**27 fewer per 1,000** (from 13 fewer to 49 more)
**90d mRS score of 6**											
1147 (6 RCTs)	Not serious	Not serious	Not serious	Serious^b^	None	⨁⨁⨁◯ Moderate	93/563 (16.5%)	99/584 (17%)	**RR 1.00** ( 0.72 to 1.37)	176 per 1000	**0 fewer per 1,000** (from 46 fewer to 61 more)
mRS score at day 90											
687 (3 RCTs)	Not serious	Serious^a^	Not serious	Serious^b^	None	⨁⨁◯◯ Low	343	344	–	–	MD 0.58 lower(1.45 lower to 0.29 more)
Mean midline shift at 72 h											
508 (2 RCTs)	Not serious	Serious^a^	Not serious	Serious^b^	None	⨁⨁◯◯ Low	250	258	–	–	MD 1.95 mm lower (7.37 lower to 3.46 more)
Decompressive craniotomy											
486 (3 RCTs)	Not serious	Not serious	Not serious	Serious^b^	None	⨁⨁⨁◯ Moderate	78/323 (24.1%)	83/328 (25.3%)	**RR 1.05** (0.80 to 1.37)	241 per 1000	**12 more per 1,000** (from 48 fewer to 89 more)
90-day mortality											
879 (4 RCTs)	Not serious	Not serious	Not serious	Serious^b^	None	⨁⨁⨁◯ Moderate	95/433 (21.9%)	100/446 (22.4%)	**RR 0.98** (0.69 to 1.39)	219 per 1000	**4 fewer per 1,000** (from 68 fewer to 86 more)
Hypoglycemia											
1261 (6 RCTs)	Not serious	Serious^a^	Not serious	Serious^b^	None	⨁⨁◯◯ Low	22/624 (3.5%)	56/637 (8.8%)	**RR 3.49** (0.96 to 12.76)	35 per 1000	**88 more per 1,000** (from 1 fewer to 415 more)
Hydrocephalus											
167 (2 RCTs)	Not serious	Not serious	Not serious	Serious^b^	None	⨁⨁⨁◯ Moderate	16/82 (19.5%)	27/85 (31.8%)	**RR 1.65** (0.97 to 2.81)	195 per 1000	**127 more per 1,000** (from 6 fewer to 353 more)

RCT: randomized controlled trial; CI: confidence interval; MD: mean difference; RR: risk ratio; mRS: modified Rankin Scale

^**a**^Wide variance of point estimates across studies

^**b**^Wide 95% confidence intervals, which include clinically important differences

## Discussion

The present systematic review and meta-analysis assessed the efficacy and safety of glibenclamide in patients with stroke, including acute ischemic stroke and acute subarachnoid hemorrhage. Our meta-analysis of eight RCTs revealed no statistically significant difference between the glibenclamide and control groups in terms of attainment of excellent (mRS score 0–1) or good (mRS score 0–2) functional outcomes, reduction of poor outcomes (mRS score 3–5), or death (mRS score 6). Furthermore, the midline shift at 72 h measurement did not show a statistically significant difference between the groups. Safety analysis did not report any significant rise in serious adverse event risk, hypoglycemia, or decompressive craniectomy risk, although a numerical trend for more hypoglycemia was observed in the glibenclamide group. These findings indicate that despite its mechanism and established effect on surrogate markers, the clinical benefit of glibenclamide is still unclear. The majority of the included RCTs in our meta-analysis assessed the mean mRS score at 90 days. In alignment with our results, all three studies showed no significant difference between the glibenclamide and placebo groups. All the RCTs that assessed the proportions of patients achieving various functional outcomes—excellent functional outcome, good functional outcome, poor functional outcome, or death—showed no significant difference between the two groups as well [1, 11, 26].

Of specific interest is that our results of no difference in all functional outcomes are in line with the findings of some landmark RCTs. For instance, the CHARM trial found no improvement in functional outcomes at 90 days, although there was some evidence that midline shift reduction was improved. Likewise, the GATE-ICH trial failed to demonstrate a significant decrease in poor functional outcomes at 90 days despite achieving a decrease in perihematomal edema. The absence of a reduction in mortality rates seen in our study is consistent with the findings from the CHARM and GAMES-RP trials, neither of which provided evidence for a reduction in mortality. Our observation of no difference in the risk rates of hypoglycemia and decompressive craniectomy is also consistent with the observations in the CHARM and GAMES-RP trials, in which the risk of these outcomes was not different in the glibenclamide and control groups. However, hypoglycemia incidence after sensitivity analysis was significantly higher in the glibenclamide group. Glibenclamide is thought to improve neurological outcomes by inhibiting novel cation channels, known as SUR1-TRPM4. The level of these channels increases significantly following cerebrovascular injury, increasing sodium influx, which culminates in cerebral edema. Therefore, glibenclamide alleviates cerebral edema and contributes to improving neurological outcomes [10, 27, 28]. In preclinical studies, it was demonstrated that glibenclamide has the potential to reduce cerebral swelling and preserve neurological function more effectively than decompressive craniectomy [29]. However, it has been challenging to translate research into clinical practice, as evidenced by the heterogeneous results among included RCTs.

In acute subarachnoid hemorrhage, brain edema is a significant contributor to mortality due to early ischemic brain injury and blood–brain barrier disruption [30, 31]. Inhibition of the SUR1-TRPM4 channel using glibenclamide has been shown to possibly reduce cytotoxic and vascular brain edema, but global functional outcomes are unclear. The phase 3 CHARM trial [11], for example, did not demonstrate reduced disability at 90 days despite some findings of less midline shift. In the GATE-ICH trial [26], glibenclamide reduced perihematomal edema volume but failed to significantly improve the distribution of functional outcomes at 90 days. Similarly, our meta-analysis results also failed to show a significant difference in functional outcome or mortality. The heterogenous pooled mRS score in our analysis was best resolved by excluding the study by Sheth et al. [11]. Perhaps the biggest obstacle in determining the efficacy of glibenclamide is the heterogeneity in study design, including differences in routes of administration (intravenous vs. oral), dosing schedules, and when treatment was started. The patients who participated in studies by Feng et al. [1], Lin et al. [2], Zhao et al. [26], and Huang et al. [9] had glibenclamide administered via oral tablets, whereas those in the studies by Sheth et al. [10, 11] had glibenclamide administered intravenously. Oral glibenclamide tablets, in contrast to intravenous treatment, face challenges related to absorption in the gastrointestinal tract [2]. Furthermore, the intervention timing differed vastly between studies, occurring anywhere from a few hours to several days after onset, which may impact the efficacy of glibenclamide for cerebral edema alleviation. For instance, in the GAMES-RP trial, intravenous glibenclamide was initiated within 9 h of stroke onset [10], whereas in the GATE-ICH trial, oral glibenclamide was started within 72 h [26]. Zhao et al. (GATE-ICH trial) demonstrated that the efficacy of glibenclamide in reducing edema was significant when administered within 24 h of onset but not when given later [26]. This finding suggests that early administration may be crucial for maximizing the therapeutic effect on brain edema in patients with ICH.

Adverse effects related to glibenclamide were mainly connected with hypoglycemia, particularly at higher doses. Although the trials used a protocol for glucose monitoring to manage low blood glucose levels, the risk of hypoglycemia remains a major limitation, especially in nondiabetic patients. This makes the need for individualized dosing strategies and careful monitoring in future studies essential. Regarding our meta-analysis, the heterogenous pooled events of hypoglycemia were best resolved by excluding the study by Huang et al. [9]. This can be attributed to the variations of hypoglycemia definitions introduced in the included RCTs. For instance, Feng et al. [1], Lin et al. [2], and Huang et al. (SE-GRACE) [9] set a threshold of ≤ 3.9 mmol/L, whereas Sheth et al. (CHARM) [11] and Sheth et al. (GAMES-RP) [10] set a threshold of < 3.1 mmol/L. This subtle variation in definitions might have affected our pooled analysis and introduced the significant heterogeneity observed in our analysis (*I*^2^ = 73%). It was hypothesized that glibenclamide could reduce the need for decompressive craniectomy by alleviating cerebral edema and lowering intracranial pressure. However, our meta-analysis did not support this assumption, as there was no statistically significant difference in decompressive craniectomy rates between the glibenclamide and control groups. According to the GAMES-RP trial, it failed to demonstrate a reduction in decompressive craniectomy despite being designed with the expectation that intravenous glyburide would decrease surgical intervention based on preliminary data from the GAMES-Pilot trial and similar patient populations [10]. A possible explanation is that GAMES-RP included patients up to 80 years old, whereas decompressive craniectomy is rarely performed in individuals older than 60, potentially confounding the results [10]. Moreover, there was a higher number of decompressive craniectomies in the intravenous glyburide group compared to the placebo group at 90 days, which may have influenced the observed reduction in mortality [10]. Nevertheless, patients treated with intravenous glyburide—regardless of whether they underwent decompressive craniectomy—had lower mortality rates than those receiving placebo [10]. Sensitivity analyses revealed comparable hazard ratios for death in both unadjusted and adjusted models for decompressive craniectomy, suggesting that the mortality benefit was independent of the surgical procedure [10]. However, the potential contribution of decompressive craniectomy to the observed mortality reduction cannot be entirely ruled out [10].

Our meta-analysis stands out in that it included all the available RCTs up to this study’s date, ensuring a well-conducted and up-to-date analysis of glibenclamide. We conducted a risk of bias assessment using RoB 2 and evaluated the certainty of evidence using the GRADE approach. Furthermore, we assessed publication bias via the LFK index and Doi plots in our primary outcomes. However, some limitations should be noted. Firstly, there was significant heterogeneity among the included studies in terms of patient populations, dosing regimens, and outcome measures. Second, combining studies with intravenous and oral administration might have brought about heterogeneity in therapeutic effects. Third, the evidence quality for specific outcomes was reduced by imprecision and heterogeneity, as indicated by our GRADE evaluation. Finally, the total sample size across included RCTs was relatively small, which may have limited the statistical power of some comparisons and potentially obscured clinically meaningful differences. Therefore, we recommend future trials to optimize the dosing strategy to balance efficacy against safety, particularly in terms of hypoglycemia management. In addition, we recommend the identification of patient subgroups most likely to gain benefit from glibenclamide. It would also be of interest to investigate synergistic effects between glibenclamide and other neuroprotective agents, such as minocycline, edaravone, or citicoline, which may offer complementary mechanisms, including antiinflammatory and antioxidant properties. Furthermore, although the primary outcome (mRS score) was generally consistent across studies, differences in the timing of administration, routes of administration, and definitions of safety outcomes (e.g., hypoglycemia thresholds) might contribute to heterogeneity. Greater standardization of outcome definitions, monitoring protocols, and treatment timing in future studies will help improve comparability and reduce variability.

## Conclusions

This meta-analysis revealed that glibenclamide, despite its mechanistic rationale in reducing cerebral edema, did not significantly enhance functional outcomes or decrease mortality in patients with acute ischemic stroke or subarachnoid hemorrhage. The interesting increased risk of hypoglycemia, after our sensitivity analysis, remains a major safety concern. Heterogeneity among trials, especially regarding administration routes and timing, may have influenced the outcomes. Although preclinical studies suggest potential neuroprotective benefits, translating these effects into clinical practice remains challenging. Future research should focus on optimizing dosing strategies, exploring potential synergistic therapies, and identifying patient subgroups most likely to benefit from glibenclamide therapy.

## Supplementary Information

Below is the link to the electronic supplementary material.

Supplementary file1 (DOCX 562 kb)

## Data Availability

All data generated or analyzed during this study are included in this published article and its supplementary file.
