# Supplementary material for: The Efficacy and Safety of Glibenclamide in Improving Cerebral Edema and Neurological Outcomes in Stroke: a GRADE-Evaluated Systematic Review and Meta-analysis with Subgroup Analysis
Source: Neurocrit Care. 2025 Jul 8;43(3):1013–32. doi: 10.1007/s12028-025-02311-3 (PMC12647293; doi:10.1007/s12028-025-02311-3)
Supplement: Supplementary file 1 — Supplementary file1 (DOCX 562 kb) [file 12028_2025_2311_MOESM1_ESM.docx]

| **Databases** | **Search strategy** |
| --- | --- |
| **PubMed, Web of Science, and Scopus** | (“cerebral edema" OR "Brain edema" OR "Subarachnoid hemorrhage” OR "Brain swelling" OR "hemispheric infarction" OR Infarction OR Stroke OR "Ischemic stroke" OR "Intracerebral hemorrhage" OR "Cerebrovascular accident") AND (Glibenclamide OR Glibenclamid OR Glybenclamide OR Glyburide) |

Supplementary Table 1: Search strategy


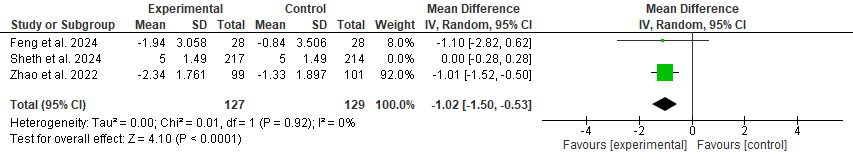

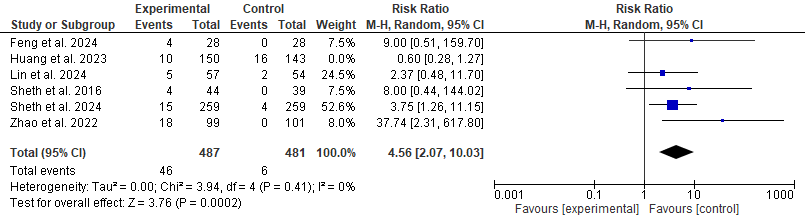


**A**

**B**

**Supplementary Figure (1)** Sensitivity analysis of **A)** mRS score at 90 days by excluding Sheth et al. 2024; **B)** Hypoglycemia adverse event by excluding Huang et al. 2023


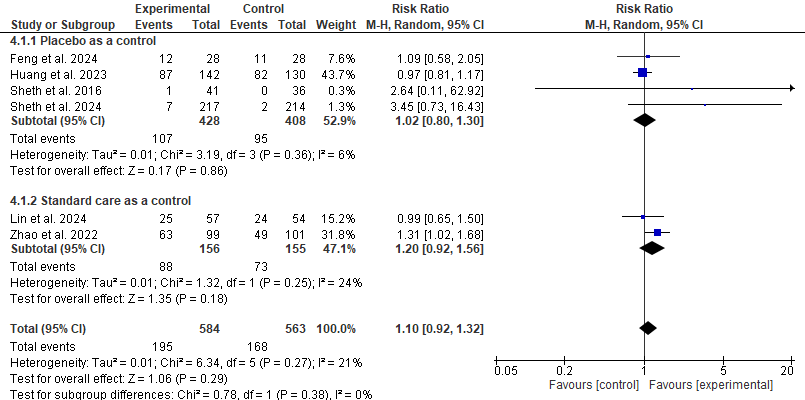

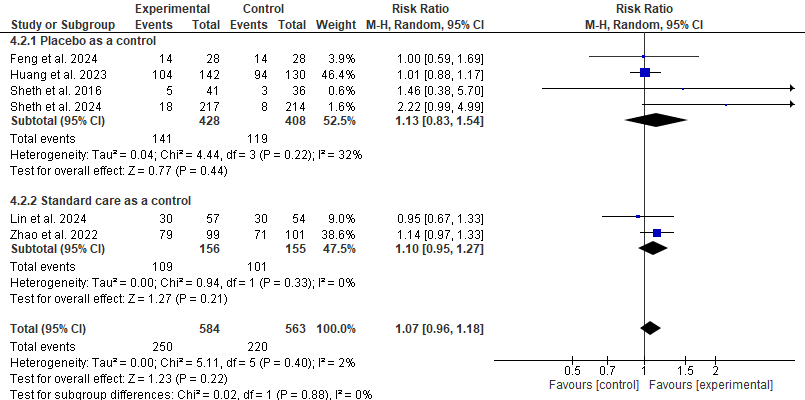


**A**

**B**


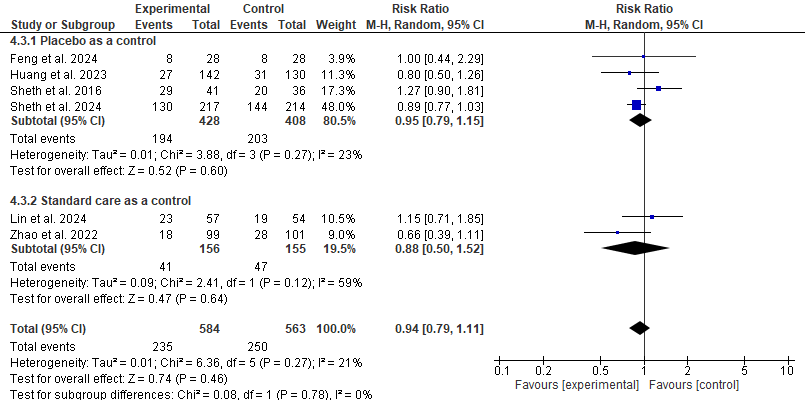

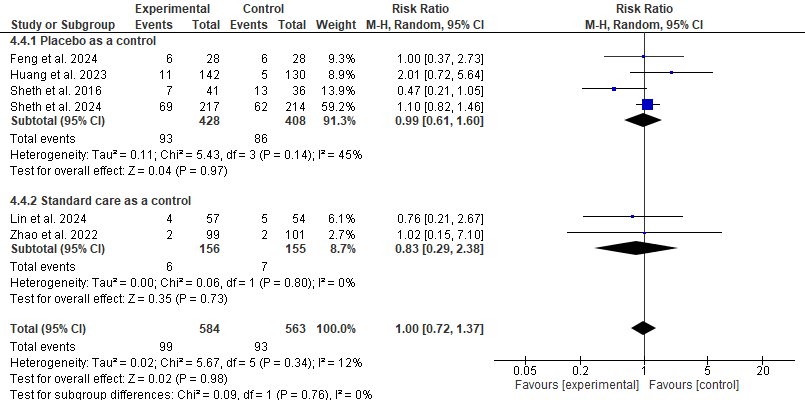


**D**

**C**

**Supplementary Figure (2)** Subgroup analysis with control group in terms of **A)** Excellent functional outcome (90d mRS score of 0-1); **B)** Good functional outcome (90d mRS score of 0-2); **C)** Poor functional outcome (90d mRS score of 3-5); **D)** Death (90d mRS score of 6)


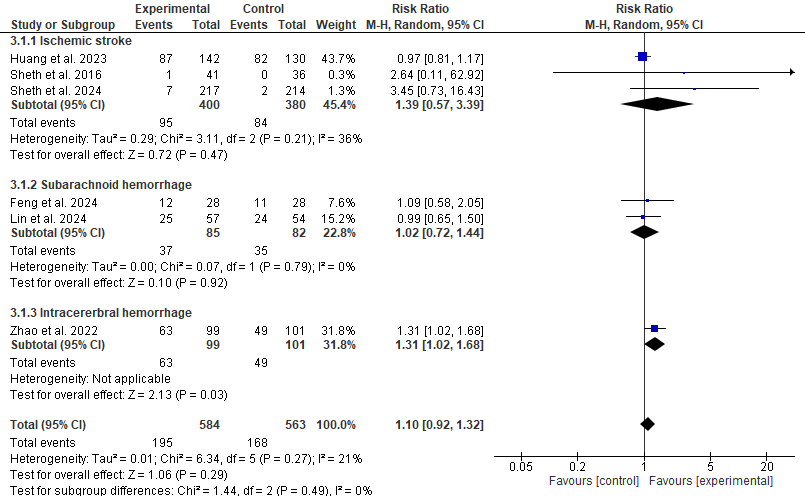

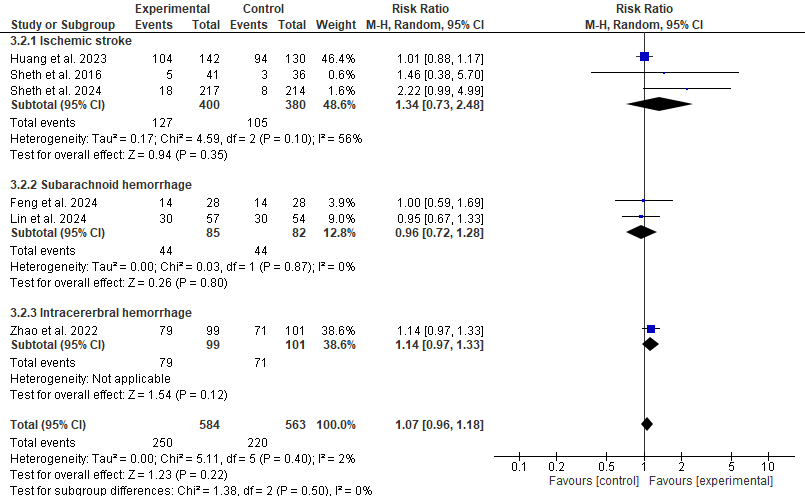


**A**

**B**


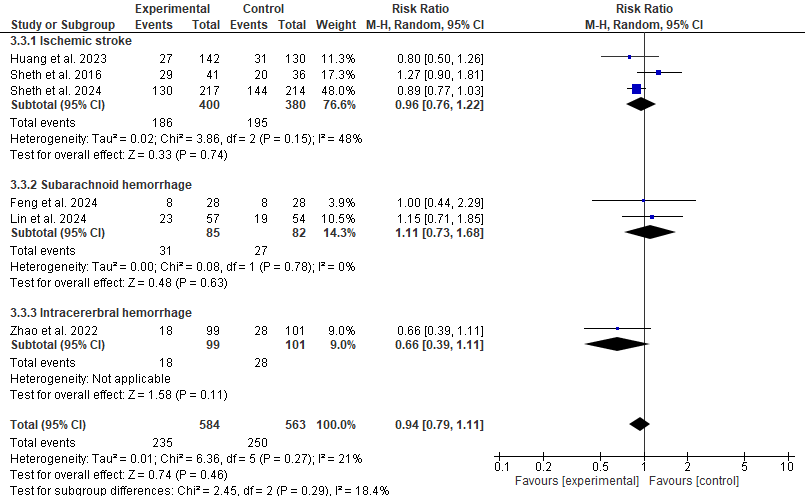

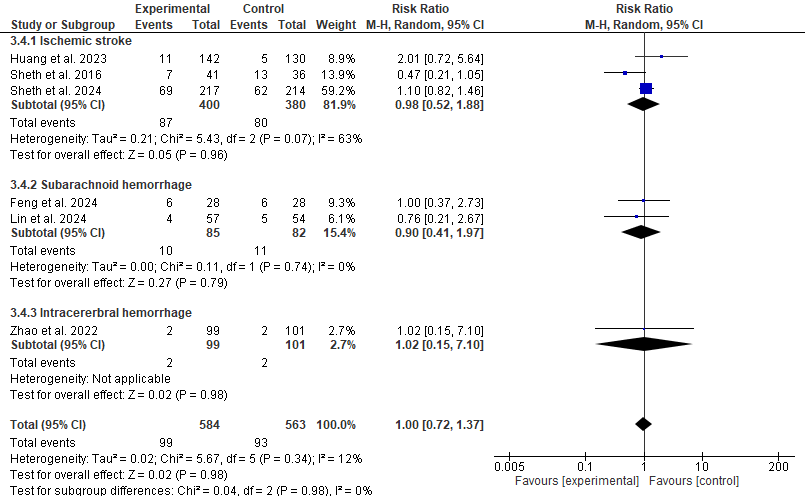


**D**

**C**

**Supplementary Figure (3)** Subgroup analysis with cerebrovascular accident (stroke) type in terms of **A)** Excellent functional outcome (90d mRS score of 0-1); **B)** Good functional outcome (90d mRS score of 0-2); **C)** Poor functional outcome (90d mRS score of 3-5); **D)** Death (90d mRS score of 6)
